# Hendrick County Medical Society, Indiana

**Published:** 1855-01

**Authors:** Henry G. Todd, J. Joel Todd

**Affiliations:** Pres.; Sec.


### MEDICAL NEWS.

*Hendricks County Medical Society, Indiana.*

This Society was organized by a called meeting of the Physicians of the County and vicinity, held at Danville, Hendricks Co., Indiana, April 29th 1854. The following are the officers for the year ending April 1855.

*President*—Henry G. Todd, M D., of Danville.

*Vice President*– Wilson Lockhart M.D., of Plainfield.

*Secretary*—J. Joel Wright, M.D., of Monrovia, Morgan Co.

*Corresponding Secretary*—Dr. L. Howard Kennedy, of Belleville.

*Treasurer*—Henry Cox M.D., of Danville.

*Censors* { Dr. Thomas B Harvey, of Plainfield.
" { Dr. Bradley Bartholemew, of Danville.
" { Henry H. Moore, M.D., of Brownsbury.

The *Constitution* of the Society provides, for the election of the foregoing officers by ballot annually—It provides, that, any regular and reputable practitioner of medicine may become a member: it provides, that, any distinguished literary gentleman may become an honorary member: it provides for the formation of a library, and a cabinet of specimens. The *By-Laws* provide, that the regular meetings of the society shall be held on the *third Tuesday* of the months of *January*, *April*, *July* and *October*, of each year; the April meeting being the annual meeting: they provide that, the President shall appoint at every meeting at least one person to write and deliver a dissertation at the next; they provide that every member shall have the privilege of reporting at any regular meeting, such cases (that have come under his own observation) as he may deem important: they provide that each member shall keep a faithful record of each important case of which he treats, and present the same to the society at the first stated meeting in each year, &c., &c.

MEMBERS.

| | |
|---|---|
| Henry G Todd, | Wilson Lockhart, |
| Henry H. Moore, | Henry Cox, |
| Thomas P. Seller, | B. Bartholomew, |
| Risdon C Moore, | J. Joel Wright, |
| Leroy H. Kennedy, | D J. Depew, |
| Thomas B. Harvey, | David Todd, |
| J. A Cominger, | W. Foster Harvey, |
| D. Hutchinson, | Wm. Matthews, |
| A. M. Reagon, | J. L. Green. |

*Extract* from the proceedings of the 4th regular meeting:

BELLVILLE, 10th Month (Oct.) 17th, 1854.

At 11 o'clock A. M., the Society met at Masonic Hall. The minutes of the preceding meeting were read and approved. On motion of Dr. Wright the order requiring the dissertations to be read in the forenoon session, was suspended. Then adjourned until 1 o'clock P. M.

AFTERNOON SESSION.

At 1 o'clock P. M., the members convened. Members present:

Drs. H. G. Todd, Bartholomew, Lockhart, Cominger, Matthews, T. B. Harvey, Cox, Kennedy, and Wright.

A Dissertation was read by Wm. Matthews M.D., on "The Varieties and Complications of the Typhoid Fever of the Interior of Indiana."

A protracted and highly interesting debate was then had, upon the subject of "Typhoid Fever," in which most of the members participated.

On motion of Dr. Cominger the discussion was brought to a close, and the Society proceeded to other business.

Dr. Kennedy, on behalf of the committee on Publication, submitted the following report—*i. e.*

We the committee on Publication of Constitution, By-Laws &c., have furnished the Society with two hundred copies of the same, for which we agreed to pay twenty-three dollars—three dollars more than we were authorized by the Society, we ask the society to pay the *extra* three dollars,

L. H. Kennedy,

H. Cox,

J. J Wright,

} Committee.

Ordered that the treasurer pay 23 dollars to the committee on publication, to defray the expenses of printing two hundred copies of the Constitution, By-Laws &c.

The censors presented in due form the name of David Hutchison, M.D., as an applicant for membership, recommending his election.

He was elected accordingly.

It was voted that 6 copies of the Constitution, By-Laws, &c., be presented to each member of the Society, in addition to those they had already received.

The President appointed David Hutchison M. D., to read a Dissertation at the next meeting.

Dr. R. C. Moore not being present, on motion he was requested to read his Dissertation at the next meeting.

Then adjourned to meet at Plainfield, at the regular time in course.

HENRY G. TODD, M. D., Pres.
J. JOEL TODD, M. D., Sec.

